# Inhaled methoxyflurane (Penthrox) for analgesia in trauma: a systematic review protocol

**DOI:** 10.1186/s13643-021-01600-0

**Published:** 2021-02-03

**Authors:** Michael M. Eager, Grant S. Nolan, Kathryn Tonks, Anoopama Ramjeeawon, Natalie Taylor

**Affiliations:** 1 Academic Department of Military General Practice & Primary Care, Research and Clinical Innovation, Royal Centre for Defence Medicine, HQ Joint Medical Group, ICT Centre, Vincent Drive, Edgbaston, Birmingham, B15 2SQ UK; 2 Department of Anaesthetics and Critical Care, Queen Elizabeth Hospital, University Hospitals Birmingham NHS Trust, Mindelsohn Way, Edgbaston, B15 2TH UK; 3 Division of Surgery & Interventional Science, University College London, Royal Free Hospital, Pond Street, London, NW3 2QG UK; 4 Department of Plastic and Reconstructive Surgery, Whiston Hospital, St. Helens and Knowsley Teaching Hospitals NHS Trust, Warrington Road, Prescot, Merseyside, L35 5DR UK

**Keywords:** Methoxyflurane, Penthrox, Systematic review, Pain, Injury, Trauma, Analgesia, Pre-hospital, Emergency

## Abstract

**Background:**

More than 75% of patients presenting to the Emergency Department are suffering symptoms of pain. Despite this, 67% will not receive any analgesia. Methoxyflurane is a fluorinated hydrocarbon gas which has analgesic properties when inhaled. Penthrox is a methoxyflurane autoinhaler recently licenced in Europe. Its ease of administration, safety, and fast onset of action make it of particular relevance to emergency medicine. Additionally, outside the hospital, it has the advantage of increased temperature stability and portability over current standard care. New evidence of its efficacy is emerging; however, currently, its use in Europe is not widespread. The objective of this study will be to systematically evaluate the evidence on inhaled methoxyflurane to determine if it is a superior analgesia in the acute trauma setting.

**Methods:**

We designed and registered a study protocol for a systematic review and meta-analysis on randomised controlled trials, comparing inhaled methoxyflurane and either placebo or standard care. A comprehensive search will be conducted from database inception onwards in MEDLINE, Embase, and the Cochrane CENTRAL database, concurrent with a search of the grey literature for other relevant studies, including clinical trial databases. Only randomised controlled trials will be included. No limitations will be imposed on publication status or language of publication. The primary outcome will be mean difference in patient-reported pain at time points within the first 30 min of administration. Secondary outcomes will be mean difference in time to clinically significant pain relief and relative risk of adverse effects. Two reviewers will independently screen all returned studies and collect data. Disagreements will be resolved through discussion or referral to a third reviewer. Individual study methodological quality will be appraised using an appropriate tool. If feasible, we will conduct a random effects meta-analysis; if this is not possible, we will construct a narrative synthesis.

**Discussion:**

This systematic review will summarise the best available evidence and definitively establish if inhaled methoxyflurane is a superior analgesia to standard care in the acute trauma setting. This knowledge will directly impact emergency care in the UK and worldwide and may require amendments to European pain relief guidelines.

**Systematic review registration:**

PROSPERO CRD42020189119.

**Supplementary Information:**

The online version contains supplementary material available at 10.1186/s13643-021-01600-0.

## Background

Methoxyflurane (the active ingredient in Penthrox) is a fluorinated hydrocarbon initially licensed as an inhaled anaesthetic agent in 1962 before reports of nephrotoxicity caused it to fall out of favour in the late 1970s; it was voluntarily removed from the market in 2001 [1–3]. However, at lower doses, it has properties as an analgesic and a self-administered methoxyflurane inhalator was marketed as the Analgizer in the 1970s. Whilst the Analgizer itself was withdrawn, methoxyflurane autoinhalers have been extensively used in Australia since 1975, are first line for moderate-to-severe analgesia in several Australian ambulance services [1], and are recommended by the Australian and New Zealand College of Anaesthetists pain management guidelines [4]. It has come into vogue in Europe relatively recently following the licencing of Penthrox, a methoxyflurane autoinhaler marketed in Europe by Mundipharma and in the UK by Galen [5]. As a result, there have been several large studies performed in the last 6 years [6–9]. Along with most anaesthetics, the mechanism of action of methoxyflurane is unclear [10].

Proponents of inhaled methoxyflurane argue that it could help minimise oligoanalgesia in the emergency department due to fewer barriers to administration compared to other medications available such as nitrous oxide, opioids, or ketamine [1, 11]. This is particularly significant as pain makes up 75–80% of all emergency department attendances [12] and yet in large-scale European studies two thirds of patients presenting with pain do not receive any analgesia [13, 14].

Other studies suggest methoxyflurane provides quicker onset of pain relief than current standard of care given the same time of provision [7, 8]. There is also evidence that it could provide a more stable, safe, and easy form of pain relief in austere or pre-hospital environments [15].

Critics have cautioned that it does not provide enough benefit over normal practice to justify its uptake [16]. There are concerns regarding potential occupational health risk to healthcare workers (particularly in the enclosed spaces found in the pre-hospital environment) [17], although this risk mitigated with the addition of an activated charcoal filter [11, 18]. Historically, there were concerns that nephrotoxicity seen at anaesthetic doses might be seen at analgesic doses, although this does not appear to be true [3].

Alternatives to inhaled methoxyflurane are morphine and Entonox (inhaled nitrous oxide) [11]. Morphine is considered the gold-standard for pain relief but can cause dependency and requires monitoring due to respiratory depression. Furthermore, the evidence suggests this leads to low starting doses and long periods of inadequate analgesia [12, 19]. Entonox equipment is bulky which delays its administration in the emergency department [11] and makes it impractical pre-hospital [15, 20].

The aim of this study will be to systematically evaluate the evidence and determine if inhaled methoxyflurane is a superior analgesia to standard care in the acute trauma setting.

## Methods

This protocol has been registered with PROSPERO international prospective register of systematic reviews (registration number CRD42020189119) and has been reported in accordance with the Preferred Reporting Items for Systematic Reviews and Meta-Analysis Protocols (PRISMA-P) 2015 statement [21]. The PRISMA-P checklist for this study is included in Additional file 1. The methodology of this review will be according to the Cochrane Handbook for Systematic Review of Interventions [22]. The final review will be reported following the PRISMA statement [23]. Should any changes to the protocol prove necessary these will be included in the final publication and updated on PROSPERO detailing the nature of the change, the date, and the rationale.

### Information sources and search strategy

The primary sources will be a structured search from inception onwards of major electronic databases: MEDLINE, Embase, and the Cochrane CENTRAL database. We will further search the grey literature for other possible relevant studies, including OpenGrey and clinical trial databases (ClinicalTrials.gov, ISRCTN registry, clinicaltrialsregister.eu, and Australian and New Zealand Clinical Trials Registry). We will perform hand-searches of reference lists of included studies. The literature searches will be designed and conducted by the review team which includes two experienced health information specialists. The search will be performed in English and any relevant non-English articles will be translated.

The search will include a broad range of terms and keywords related to methoxyflurane, Penthrox, trauma, pain, and study design (based on the Cochrane Handbook randomised controlled trials [RCT] search guideline) [22]. The search strategy for MEDLINE is included as Additional File 2.

### Study selection

After removing duplicates, search results will be screened by two reviewers independently using titles and abstracts against pre-specified inclusion and exclusion criteria. All included articles at this point will the undergo a full text review and the data will be extracted. This will again be performed independently by two reviewers. Any discordance at any stage will be resolved through discussion or referral to a third reviewer if required. The screening process will be performed in Rayann [24], a web-based application for systematic review. The reason for exclusion of records will be recorded at each stage. Records will be kept of each stage and a PRISMA flow-chart published with the review**.**

### Eligibility criteria

Studies will be included or excluded based on the following criteria, sub-divided into population, intervention, comparator, outcome measures, and study design. We will not exclude based on publication status, language, or date of publication.

### Participants/population

We will include clinical trials on patients of all ages with traumatic pain within the acute setting—specifically the pre-hospital environment (including transport) and the emergency department. We will exclude studies utilising methoxyflurane for procedural or obstetric analgesia.

### Interventions

In order to maintain relevance to the newly licensed Penthrox autoinhaler, we will include only studies which offer self-administered methoxyflurane at doses similar to those Penthrox is currently licenced for (3 ml/h and max 6 ml/day, or 0.59 MAC-hours [minimum alveolar concentration hours]) [25]. We will exclude any studies using doses above 2.0 MAC-hours, the level at which nephrotoxicity has been reported in humans [3].

### Comparators

It has been reported there has previously been a paucity of controlled studies on methoxyflurane using active comparators [20]. As a result we will include studies using all comparators, including placebo with rescue medications.

### Outcome measures

The primary outcome will be mean difference in patient-reported pain relief using any validated pain scale (such as visual analogue scale [VAS] or visual numerical rating scale [VNRS]) from time of administration up to 30 min after. The secondary outcomes will be mean difference in time to first clinically significant pain relief from time of administration and relative risk of adverse events.

### Study designs

We will only include RCTs. We will exclude non-randomised trials including cohort studies, letters, reviews, cases series, case reports, and subgroup analyses of other studies.

### Settings

Studies will be included if they take place in the emergency department or in the pre-hospital setting. Studies taking place outside of this, such as in theatre, will be excluded.

### Data extraction

All full-text articles identified as appropriate for the review will have their data extracted independently and in duplicate by two reviewers using a standardised data extraction sheet with the following headings:

1. Study characteristics
  - Lead author, year, title, study design, blinding, and pain scale used
  - Population
    I Country of study, location, and inclusion and exclusion criteria
  - Intervention dose and frequency
  - Comparator including definition of standard of care if included
  - Primary and secondary study outcomes

## Results

(f) Number screened, number included, number of drop outs, and length of follow up
(g) Demographics (number, age, and gender of control and treatment arms, mean and standard deviation of each where appropriate)
(h) Mean/median and standard deviation of pain scores in control and treatment arm at baseline and at 5, 10, 15, 20, and 30 min, as well as any other times added
(i) Mean time to reported pain relief in control and treatment arm if recorded including confidence intervals
(j) Adverse effects recorded in control and treatment arm

In the event of missing data, we will contact the authors via email to request access to their data as this has the highest rate of response [26]. As evidence shows that 30% of authors are uncontactable [27] and that repeated attempts to contact them do not increase success [26], we will only attempt to contact authors once. If full data is not obtainable, studies with partial data will be included in the synthesis if appropriate and missing data will be imputed from reported outcomes as possible in line with the Cochrane handbook [22].

### Assessment of risk of bias of included studies

We will use the Cochrane RoB (Risk of Bias) 2 tool [28] to assess risk of bias for individual RCTs. They will be scored independently and in duplicate with any disagreements being resolved through discussion and referral of a third independent review if required. The results of the risk of bias tool will be used in a sensitivity analysis to ensure studies judged to be at ‘high’ risk of bias do not affect the robustness of our results in any subsequent meta-analysis.

### Data analysis

To address the main review question, data will be synthesised to establish whether methoxyflurane is superior to standard care for analgesia in acute trauma. The data from included study will be used to build evidence tables for an overall description of included studies for methoxyflurane in the trauma setting. This will include study characteristics, context, participants, outcomes, and findings. If the same pain scales have been used, then we will calculate the mean difference with 95% confidence intervals of inhaled methoxyflurane vs standard care/placebo. In the event that different scales have been used we will calculate standardised mean difference with 95% confidence intervals. The results of this will be presented in a forest plot. This will be undertaken in RevMan [29].

To determine the extent of variation between selected studies, tests of heterogeneity will be performed. Inter-study heterogeneity will be assessed visually using the forest plot. Statistical heterogeneity will be quantified statistically using three tests. The *I*^2^ statistic will be used and the result will be interpreted using the definitions in the Cochrane Handbook for Systematic Reviews of Interventions [22]. Additionally, the Chi^2^ and Tau^2^ statistic will be used where a *p* value < 0.10 will be deemed as statistically significant for heterogeneity. Any sources of heterogeneity will be explored using subgroup analysis.

If two or more comparative studies with an absence of clinical heterogeneity are identified by the systematic review, then we will perform a random effects meta-analysis. This approach is appropriate given it is likely that the true effect size varies from study to study and these follow a normal distribution.

### Additional analysis

If sufficient studies are identified and data points are available, potential sources of heterogeneity will be investigated further by subgroup or meta-regression analyses. We plan to conduct analysis to establish the relative risk of adverse events presented in the trials. Subgroup analysis will be considered on trials vs placebo with rescue medicine and trials vs active comparators. We will use the grading of recommendations assessment, development, and evaluation (GRADE) working group methodology [30] to assess our confidence in the cumulative evidence and will include a full explanation of our rationale. If quantitative synthesis is not appropriate for any reason (e.g. clinical heterogeneity) a narrative synthesis undertaken according to synthesis without meta-analysis (SWiM) guidelines [31].

Sensitivity analysis will be performed on any studies with imputed values and studies judged to be at high risk of bias to ensure the robustness of our results.

### Meta-biases

If there are 10 or more studies with the same outcome, publication bias will be assessed by inspecting a funnel plot for asymmetry and, if appropriate, using Egger’s test [32].

## Discussion

Pain as a symptom is both ubiquitous and difficult to manage, particularly early in the patient care pathway when other considerations can seem more pressing. Early effective pain relief is something we owe our patients but with limited time it is important that we are confident in the evidence base behind our decisions, particularly when considering a change in practice. This systematic review protocol presents the method to identify and synthesise the highest quality of evidence regarding the level of pain relief provided by inhaled methoxyflurane in traumatic pain in comparison to current practice, the first to do so since the publication of the recent European RCTs. This in turn will help inform the discussion on the more widespread introduction of methoxyflurane autoinhalers into current practice in Europe.

One of the main issues anticipated with this study is the difficulty in defining a clinically significant—as opposed to a statistically significant—change in a patient-reported pain scale. The literature is significantly divided on this and it is unclear whether absolute or relative changes are more valuable to patients. The absolute values proposed range from 8 to 40 mm on the VAS scale [33]. A commonly cited number for clinical significance is 13 mm [8, 15, 20], although this is disputed and some argue that a higher threshold should be used when considering a change in practice to decrease the chance of a clinical reversal [34]. However, for the purposes of ascertaining first clinically significant pain relief we will use 13 mm with the understanding that this is based on consensus rather than a clear clinical cut off.

There are some other potential limitations anticipated, particularly the paucity of higher levels of evidence comparing inhaled methoxyflurane with active comparators as previously identified [35]. We hope that the recently published European studies will rectify this short fall. We also anticipate a significant proportion of included studies will be industry sponsored which a Cochrane review found to increase the degree of efficacy of a new medication [36]. If this is the case, we will identify and discuss this in the review publication.

## Supplementary Information

**Additional file 1:.** PRISMA-P Checklist

**Additional file 2:.** MEDLINE search strategy

## Data Availability

Data sharing is not applicable to this article as no datasets were generated or analysed during the current protocol.

## Abbreviations

Embase: Excerpta Medica Database
GRADE: Grading of recommendations assessment, development, and evaluation
MAC: Minimum alveolar concentration
PRISMA: Preferred reporting items for systematic reviews and meta-analysis
PRISMA-P: Preferred reporting items for systematic reviews and meta-analysis protocols
RCT: Randomised controlled trial
RoB: Risk of bias
SoC: Standard of care
SWiM: Synthesis without meta-analysis
UK: United Kingdom of Great Britain and Northern Ireland
VAS: Visual analogue scale
VNRS: Visual numerical rating scale
