# Supplementary material for: Inhaled methoxyflurane (Penthrox) for analgesia in trauma: a systematic review protocol
Source: Syst Rev. 2021 Feb 3;10:47. doi: 10.1186/s13643-021-01600-0 (PMC7860209; doi:10.1186/s13643-021-01600-0)
Supplement: Supplementary file 2 — Additional file 2:. MEDLINE search strategy [file 13643_2021_1600_MOESM2_ESM.pdf]

Medline via PubMed Search Strategy

		Search group
1	Randomised controlled trial [pt]	**Study design**
2	Controlled clinical trial [pt]	
3	Randomized [tiab]	
4	Randomised [tiab]	
5	Placebo [tiab]	
6	Drug therapy [sh]	
7	Randomly [tiab]	
8	Trial [tiab]	
9	Groups [tiab]	
10	1 OR 2 OR 3 OR 4 OR 5 OR 6 OR 7 OR 8 OR 9	
11	Methoxyflurane [tiab]	**Intervention**
12	*Methoxyflurane	
13	Analgizer [tiab]	
14	Penthrox [tiab]	
15	Penthrane [tiab]	
16	11 OR 12 OR 13 OR 14 OR 15	
17	Analgesi* [tiab]	**Trauma or Emergency Related Pain**
18	Pain [exp]	
19	17 OR 18	
20	Traumatic injur* [tiab]	
21	Trauma [exp]	
22	Emergency department [tiab]	
23	Emergency [exp]	
24	Pre*hospital OR pre[adj1]hospital	
25	Wound* OR laceration* OR contusion* OR burn* OR dislocation* OR fracture*	
26	20 OR 21 OR 22 OR 23 OR 24 OR 25	
27	19 AND 26	
28	10 AND 16 AND 27	
